# Thymic neuroendocrine tumors in patients with multiple endocrine neoplasia type 1

**DOI:** 10.1007/s12020-022-03099-4

**Published:** 2022-06-13

**Authors:** Iina Yliaska, Heikki Tokola, Tapani Ebeling, Outi Kuismin, Olavi Ukkola, Minna L. Koivikko, Timo Lesonen, Jussi Rimpiläinen, Tuuli Felin, Eeva Ryhänen, Saara Metso, Camilla Schalin-Jäntti, Pasi Salmela

**Affiliations:** 1 Medical Research Center Oulu, Oulu University Hospital, Research Unit of Internal Medicine, University of Oulu, Oulu, Finland; 2 Department of Pathology, Cancer Research and Translational Medicine Research Unit, University of Oulu and Oulu University Hospital, Oulu, Finland; 3 Department of Clinical Genetics, Oulu University Hospital, Medical Research Center Oulu and PEDEGO Research Unit, University of Oulu, Oulu, Finland; 4 Medical Research Center Oulu, Oulu University Hospital, Oulu, Finland; 5 Department of Surgery, Oulu University Hospital, Oulu, Finland; 6 Tampere University, Faculty of Medicine and Health Technology and Tampere University Hospital, Department of Internal Medicine, Tampere, Finland; 7 Endocrinology, Abdominal Center, Helsinki University Hospital and University of Helsinki, Helsinki, Finland

## Abstract

**Objective:**

MEN1 is associated with an increased risk of developing tumors in different endocrine organs. Neuroendocrine tumors of the thymus (TNETs) are very rare but often have an aggressive nature. We evaluated patients with MEN1 and TNET in three university hospitals in Finland.

**Design/Methods:**

We evaluated patient records of 183 MEN1-patients from three university hospitals between the years 1985–2019 with TNETs. Thymus tumor specimens were classified according to the new WHO 2021 classification of TNET. We collected data on treatments and outcomes of these patients.

**Results:**

There were six patients (3.3%) with MEN1 and TNET. Five of them had the same common gene mutation occurring in Finland. They originated from common ancestors encompassing two pairs of brothers from sequential generations. The mean age at presentation of TNET was 44.7 ± 11.9 years. TNET was classified as atypical carcinoid (AC) in five out of six patients. One patient had a largely necrotic main tumor with very few mitoses and another nodule with 25 mitoses per 2 mm^2^, qualifying for the 2021 WHO diagnosis of large cell neuroendocrine carcinoma (LCNEC). In our patients, the 5-year survival of the TNET patients was 62.5% and 10-year survival 31.3%.

**Conclusion:**

In this study, TNETs were observed in one large MEN1 founder pedigree, where an anticipation-like earlier disease onset was observed in the most recent generation. TNET in MEN1 patients is an aggressive disease. The prognosis can be better by systematic screening. We also show that LCNEC can be associated with TNET in MEN1 patients.

## Introduction

Multiple endocrine neoplasia type 1 (MEN1) is an inherited autosomal dominant tumor-predisposing syndrome caused by inactivating mutations of the *MEN1* gene, which is located on chromosome 11q13 and encodes the 610 amino acid protein menin [1, 2]. The classical manifestations of the syndrome are primary hyperparathyroidism (PHPT), pituitary adenomas, and pancreatic (PNET) and duodenal (DNET) neuroendocrine tumors. In addition, also other tumors such as thymic neuroendocrine tumors (TNET) associate with MEN1 [3].

TNETs are very rare neoplasms accounting for only 0.4% of all neuroendocrine tumors and 5% of all anterior mediastinal neoplasms [4], and their age-adjusted incidence rate in the USA is 0.18 per one million persons [5]. These tumors can be sporadic, but nearly 25% of TNETs are associated with MEN1 [6]. TNETs are often aggressive and are an important cause of morbidity and mortality among MEN1 patients [7].

In the most recent 2021 World Health Organization (WHO) classification of thymic tumors, TNETs are classified into low-grade typical carcinoids (TC), intermediate-grade atypical carcinoids (ACs), and two high-grade malignancies, large cell neuroendocrine carcinomas (LCNEC) and small cell carcinomas (SCC), similarly to the classification of broncho-pulmonary neuroendocrine neoplasms (bpNEN) [8]. Due to only a few reported studies it is still uncertain whether this classification is prognostic for TNET [9]. There are recent large series of patients on the outcome of TNET [5, 10], but these studies do not contain data on the natural course of TNET in MEN1 patients, in whom only small studies with a low number of patients are available [6, 7, 11–21]. There are no randomized clinical trials and no definitive guidelines on the screening, diagnosing and treatment of TNETs in patients with MEN1. As published data is scarce, we searched for MEN1 patients characterized by TNETs within a large cohort of Finnish patients. Here, we describe the clinical characteristics, family history and underlying mutations, diagnostic work-up, histopathological characteristics, treatments and outcome of MEN1 patients with TNETs.

## Patients and methods

Patients with MEN1 were collected from patient files from the years 1985 to 2019 from three university hospitals in Finland. TNET was diagnosed according to histopathological examination of tumor tissue. All patients with TNETs had their follow-up at the Endocrine unit at the Oulu University Hospital. Standard biochemical and radiological methods were used in screening and follow-up protocols were based on current guidelines [2]. Clinical and demographic data were collected retrospectively from medical records. The study protocol was approved by the medical ethics committee of the North Ostrobothnia Hospital District and Board reviews at the three University Hospitals, Oulu, Helsinki and Tampere. Every patients had given an informed consent before the genetic testing in the departments of the clinical genetics of the participating University Hospitals. The Declaration of Helsinki was adhered to during the study.

### Mutation analysis

Mutation analysis for five of the subjects was performed and described as part of our earlier work [22] and for one case as diagnostic laboratory service using polymerase chain reaction (PCR) and DNA sequencing.

### Tumor Specimens and Immunohistochemistry

We used archival formalin-fixed, paraffin-embedded (FFPE) tissue slides and blocks of TNETs from six MEN1 patients collected between 1994 and 2015 at Oulu University Hospital, Finland. Materials consisted exclusively of resection specimens of thymic primary tumors. All cases were carefully reviewed by a pathologist (H.T.) and classified according to the WHO 2021 classification scheme [8]. Mitotic counts were determined in 10–30 high-power fields (HPF) on hematoxylin and eosin-stained sections using a Nikon Eclipse Ci-E microscope (40× objective, 10× eyepiece, field-of-view diameter of 0.625 mm, resulting in 10 HPF = 3.07 mm^2^). Only unequivocal mitoses were counted. The results are expressed as average of number of mitoses per 2 mm^2^.

If archival immunostainings were missing or technically unsatisfactory, immunohistochemistry (IHC) was performed after antigen retrieval on a BOND RX stainer platform (Leica Biosystems, Nussloch, Germany) using BOND Polymer Refine Detection (Leica Biosystems) as the detection system. All stains were performed using standard protocols. Antibodies included synaptophysin (code NCL-L-SYNAP-299, clone 27G12, dilution 1:50; Leica Biosystems), chromogranin A (code 238M-96, clone LK2H10, 1:400; Cell Marque, Rocklin, California, US), cytokeratin (code M0821, clone MNF116, 1:300; Dako Agilent, Santa Clara, California, US), and Ki-67 (code PA0230, clone K2, ready-to-use; Leica Biosystems). For analysis of Ki-67 index, representative photographs of regions of most intense labeling (hotspots) were taken at 400× magnification, printed out, and positive versus negative tumor cell nuclei were counted manually. The results are expressed in percentages by using two different definitions for a hotspot: First, an area containing at least 500 cells, as currently used for the 2019 WHO classification of gastroenteropancreatic neuroendocrine tumors (GEP-NET) [23], and second, an area of at least 0.4 mm^2^, as defined in the 2018 International Agency for Research on Cancer (IARC) and WHO expert consensus proposal for a uniform classification of neuroendocrine neoplasms (NEN) [24].

### Statistical analysis

Results are expressed as mean ± SD or median and range. Survival curves were computed by Kaplan–Meier analysis.

## Results

### MEN1 patients characterized by TNETs

Among 183 patients with MEN1 TNET was diagnosed in six cases, with a prevalence of 3.2%. Clinical findings, family histories and mutation analyses of patients with TNETs are given in Tables 1 and 2. Mutations in the *MEN1* gene were observed in all of them, and a detailed family history of MEN1 was also available in every patient.

**Table 1:** Clinical characteristics of the patients

Patient	Gender		Mutation		Age at diagnosis of MEN1	Age at diagnosis of TNET	Family history of MEN1	Family history of TNET
		code	nature	exon				
1	Male	c.157C > T	non-sense	10	28	56	+	−
2	Male	c1546insC	insertion	10	46	46	+	+
3	Male	c1546insC	insertion	10	26	31	+	+
4	Male	c1546insC	insertion	10	53	53	+	+
5	Male	c1546insC	insertion	10	36	44	+	−
6	Male	c1546insC	insertion	10	18	26	+	+

*MEN1* multiple endocrine neoplasia syndrome type 1, *TNET* thymic neuroendocrine tumor

**Table 2:** Main features of MEN1

Patient #	First presenting lesion of MEN1	MEN1 lesions					Other diseases	Smoking
		HPT	Pancreatic	Pituitary	Adrenal	Other		
1	HPT	+	−	−	+	Collagenoma of the skin	Papillary renal carcinoma	Non-smoker
2	TNET	+	−	−	+	−	Asthma, aortic stenosis	Ex-smoker
3	HPT	+	+	+	+	Lipomas, angiomyolipoma	Epilepsy, type 2 diabetes mellitus	Ex-smoker
4	TNET	+	+	−	−	Lipomas	Cerebral infarction	Non-smoker
5	HPT	+	−	−	−	−	−	Non-smoker
6	HPT	+	+	+	−	−	Type 2 diabetes mellitus, obesity, hypercholesterolemia	Ex-smoker

*MEN1* multiple endocrine neoplasia syndrome type 1, *TNET* thymic neuroendocrine tumor, *HPT* primary hyperparathyroidim

All patients characterized by TNETs were males [Table 1]. MEN1 was diagnosed at the mean age of 34.5 ± 13.1 years [Table 1]. First presenting manifestations of MEN1 are shown in Table 2. TNET was the primary presentation of MEN1 in two patients, who were brothers. During the follow-up, MEN1 was associated with PHPT in every patient, PNET was present in three patients (50%), pituitary adenoma in two patients (20%), and adrenal tumor in three patients (50%).

The mean age at presentation of TNET was 44.7 ± 11.9 years (range 25–56 years). The family history of TNET was obtained in four patients [Table 1]. TNET was observed by screening CT-examinations in 50% of the patients and in others because of chest symptoms [Table 3]. In one patient (#5), tumor screening and follow-up were delayed and irregular after MEN1 diagnosis because he was working in another part of Finland. Three patients were operated for PHPT before TNET was diagnosed. Prophylactic cervical thymectomies were not performed in any of them [Table 3]. Three patients had a positive smoking history. They were all ex-smokers at the time of TNET diagnosis [Table 2].

**Table 3:** Diagnostics and clinical features of TNET

Patient #	Symptoms	Ectopic hormone secretions	Radiological findings at diagnosis	Scintigraphy at diagnosis	P-CgA nmol/L	Previous cervical parathyroidectomy and partial thymusectomy
1	Screening	None	CT	Octreoscan positive	10,7 (<4)	–
2	Chest pain	None	CT	Octreoscan positive	2,3 (<4)	–
3	Screening	None	CT	Octreoscan negative	3 (<6)	–
4	Dyspnea, chest pain	None	X-ray	Octreoscan positive	3,8 (<4)	–
5	Cough	None	X-ray/CT	Octreoscan positive	33,9	–
6	Screening	None	CT	Octreoscan positive	4,1 (<6)	–

*TNET* thymic neuroendocrine tumor, *CT* computer tomography, *CgA* chromogranin A

Somatostatin receptor scintigraphies (SRS) (Octreoscan) were positive in five patients [Table 3]. In two patients, plasma chromogranin levels were slightly increased at the time of the diagnosis of TNET. These patients had no PNET findings [Table 3]. None of the patients showed clinical or laboratory findings indicating ectopic hormone secretion. However, in patient #5, serum IGF-1 levels were increased during the time of reoperation and remained slightly elevated for four years, but serum growth hormone was at a high normal level, pituitary SRS was negative, and no clinical signs of acromegaly were noticed.

### Family history and *MEN1* mutations

**Fig. 1:**
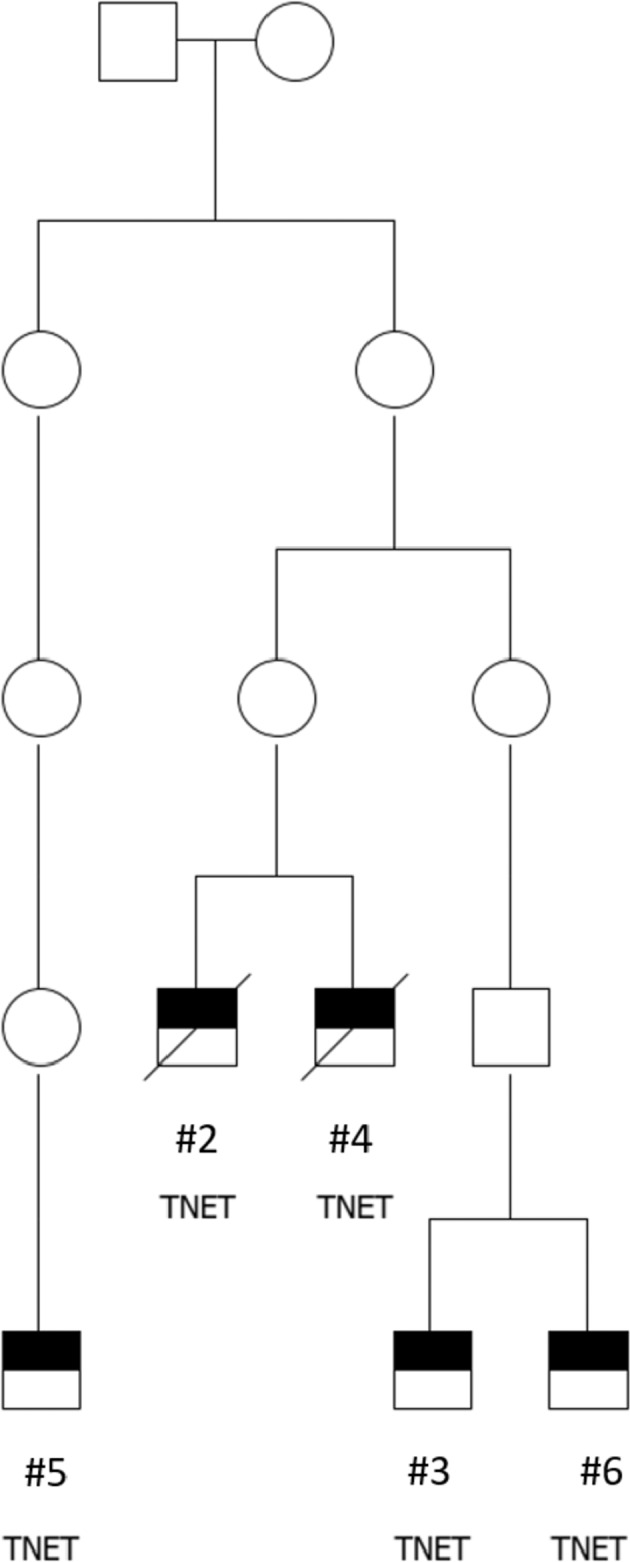
Family tree of a large pedigree of a founder mutation of MEN1 in Northern Finland. TNET-patients and their ancestors shown only

Five patients originated from common ancestors with a founder mutation in Northern Finland [Fig. 1]. In our pedigree and in the two pairs of brothers from sequential generations, the most recent generation was diagnosed with TNET at a younger age with a mean age of 28,5 years compared to the pair of the earlier generation with a mean age of 49,5 years [Fig. 1, Table 1]. The MEN1 (NM_130799.2, NP_570711.1) pathogenic variants identified in the patients were c.1579 C > T and c.1546_1547insC [Table 1].

### TNET pathological findings

**Fig. 2:**
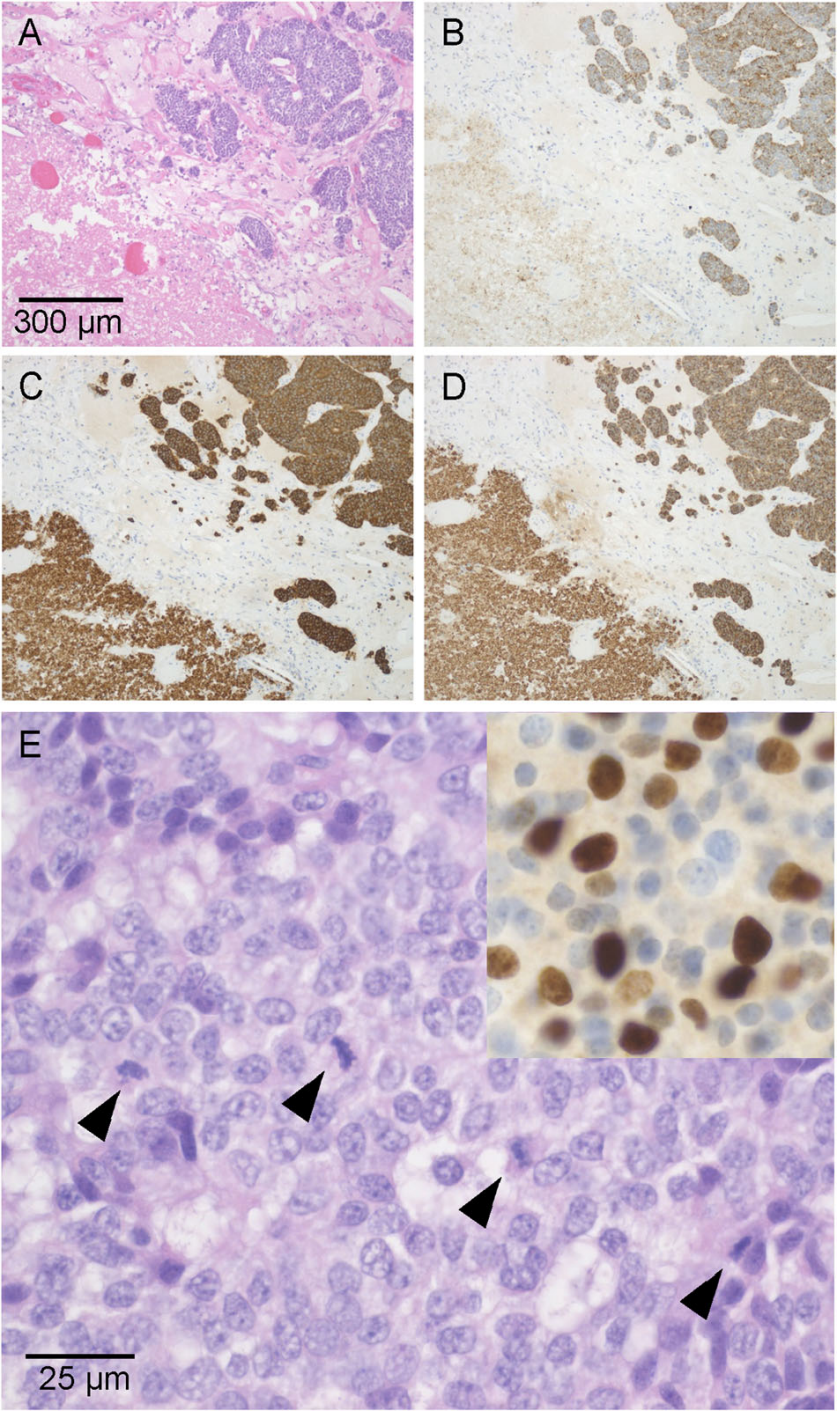
Large cell neuroendocrine carcinoma of patient #4. **A**–**D**, low-power magnification showing organoid architecture and large necrosis on bottom left. Hematoxylin and eosin **A**, cytokeratin **B**, synaptophysin **C** and Chromogranin A **D**. Original magnifications **A**–**D**: 100×. **E** solid growth with the number of mitotic figures (arrowheads) exceeding 10 mitoses per 2 mm^2^, thereby fulfilling the 2015 WHO criteria of LCNEC. Ki-67 staining in the insert on top right (hematoxylin and eosin; original magnifications: 630×)

At the time of diagnosis, the size of TNETs ranged from 14 mm to 130 mm [Table 4]. In the patients whose TNETs were diagnosed by CT screening, the size of TNET was smaller than in those with symptom driven diagnosis. All the tumors were confirmed to be chromogranin A and synaptophysin positive. Based on mitotic activity and absence/presence of necrosis, five out of six cases were classified as AC (atypical carcinoid) according to the 2021 WHO classification of thymic neuroendocrine tumors [8] [Table 4]. One patient (#4) had a largely necrotic 80 mm main tumor with only four mitoses per 2 mm^2^, and a separate 10 mm nodule with 25 mitoses per 2 mm^2^ qualifying for the 2021 WHO diagnosis of large cell neuroendocrine carcinoma (LCNEC) [Fig. 2]. The Ki-67 indices of ACs varied from 4.1 to 12.5% or from 3.6 to 9.9% depending on whether they were calculated according to the 2019 WHO classification of GEP-NETs [23] or the 2018 WHO consensus proposal [24] for a common classification framework for neuroendocrine neoplasms (NEN), respectively, whereas the Ki-67 index of the LCNEC was 16.5 or 20.3%, respectively [Table 4].

**Table 4:** Features of the TNETs

Patient#	Tumor size (mm)	Mitotic count per 2 mm^2^	Necrosis	2021 WHO classification of TNET	Ki-67 cell labeling index/500–1000 cells	Ki-67 cell labeling index/≥0.4 mm^2^	2018 WHO proposal for a uniform classification of NENs
1	50	8	Focal	AC	8.0 (734)	5.9 (4592)	NET G2
2	50	8	Diffuse	AC	5.4 (539)	4.8 (2982)	NET G2
3	Multiple small	3	Absent	AC	12.5 (691)	9.9 (4351)	NET G2
4	80	25	Diffuse	LCNEC	20.3 (939)	16.5 (5433)	NEC, large cell-type
5	130	5	Focal	AC	10.3 (864)	7.3 (4599)	NET G2
6	14	9	Absent	AC	4.1 (635)	3.6 (3855)	NET G2

Ki-67 indices are calculated in two different ways as described in Methods. The numbers of calculated cells are shown in parentheses

*WHO* World Health Organization, *TNET* thymic neuroendocrine tumor, *NEN* neuroendocrine neoplasm, *AC* atypical carcinoid, *LCNEC* large cell neuroendocrine carcinoma, *NET* neuroendocrine tumor

Primary features of the TNETs at diagnosis. In addition to current 2021 WHO classification of TNET [8], tumors are classified according to 2018 WHO proposal for a uniform classification of NENs [24]

### Treatment

TNET was operated in 5 patients by a median sternotomy and in one (#3) by a thoracoscopy [Table 5]. In two patients, TNETs were encapsulated (#1, #3). In the other patients, tumors were partially encapsulated (#4) and infiltrated locally into adjacent structures (#6) or regionally into the pleura, sternum, lung or nerve and vascular structures (#2, #5). In all patients, primary radical tumor resections were possible by removing tumors and the invaded thoracic structures. Macroscopically, resections were initially thought to be curative in 3 patients (#1, #3, #6). Distant metastases were not found at the time of the primary operation in any of the patients. Four patients underwent reoperation after the primary surgery. In one case (#2), the reoperation was done 9 months after the primary surgery due to a positive postoperative SRS result, but no tumor was found in the operation. In the other 3 patients, reoperations were done 2 to 6 years afterwards.

**Table 5:** Treatments of patients with TNET

Patient #	Primary surgery	Radiation therapy	SSA-therapy	Chemotherapy	Other treatments	Treatment of recurrence
1	12/2002	−	−	−	Interferon to renal carcinoma	−
2	5/1994	+	−	−	−	reoperation 6/1996
3	9/2011	−	−	−	−	−
4	2/1996	−	−	−	−	reoperation 1/1999
						radiation 3/1999
						interferon 5/2003
						SSA 5/2003
						chemo 2/2004
5	11/2007	−	−	−	−	reoperation 6/2013
						PRRT 10/2013 + 2/2019
						SSA 6/2014
						chemo 6/2016
						radiation 10/2016
						everolimus 2/2017
6	2/2015	−	+	−	−	reoperation 11/2015

*SSA* somatostatin analog, *PRRT* peptide receptor radionuclide therapy

Three patients received radiotherapy [Table 5], one of them (#2) after the primary operation and two others after the reoperations. Two patients received somatostatin analog therapy (SSA) [Table 5]. In one of them, SSA was used with interferon. No significant positive response was observed with these therapies, but minor palliative responses cannot be excluded, especially for serum IGF-levels. Two patients received chemotherapy [Table 5]; one of them had etoposide plus cisplatin therapy and the other temozolomide plus capecitabine therapy. No significant responses to chemotherapy were observed as tumor metastases showed progression.

Everolimus was used in one patient #5 [Table 5], and a significant stabilization in the progression of metastasis was observed during 13 months. This patient also received peptide receptor radionuclide therapy (PRRT) by lutetium-octreotate treatment, which was given twice during the follow-up. The first four treatment cycles were initiated soon after the reoperation, but the patient still progressed. The second three treatment cycles were done five years later in palliative-intent therapy. The therapeutic role of PRRT was difficult to assess, but cannot be excluded in the rather long course of the disease in this patient.

### Survival

The median follow-up of the patients was 7.1 years (range 8–25 years) [Table 6]. Three patients died during the follow-up. One of them (# 1) died of renal papillary carcinoma at the age of 60 years. At the time of his death, there were no signs of any recurrent primary TNET or metastases. In one other patient (# 2), after a follow-up of 62 months regional and distant metastases were observed and further treatments were planned, but the patient died of myocardial infarction before these were started. The third (#4) patient died of TNET after a follow-up of 102 months. The other three patients were alive at the end of the follow-up of 8.4 years.

**Table 6:** Outcomes of patients with TNET

Patient #	Duration of follow-up of TNET mo	Curative result	Time from primary to first recurrence mo	Metastases during follow-up	Cause of death
1	46	+	−	−	renal carcinoma
2	62	−	22	Bone, mediastinum, lung, kidney	cardiac cause
3	99	+	−	−	alive
4	102	−	31	Mediastinum, upper thorax	TNET
5	145	−	65	Pleura, mediastinum, bone, kidney, retroperitoneum	alive
6	58	−	7	−	alive

*TNET* thymic neuroendocrine tumor

**Fig. 3:**
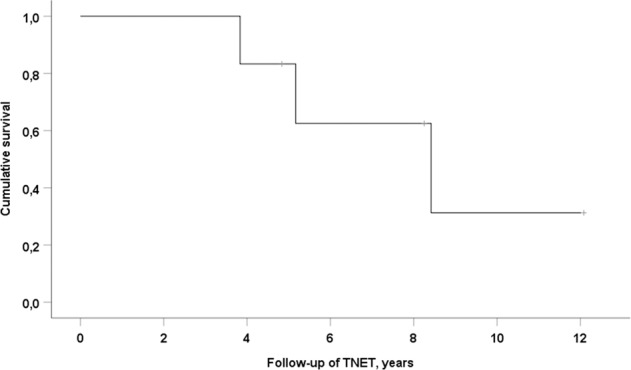
Kaplan–Meier curve showing overall survival of the MEN1 patients with TNET

The Kaplan–Meier curve is shown in Fig. 3. The 5-year survival of the TNET patients was 62.5% and 10-year survival 31.3%.

## Discussion

According to the current 2021 WHO classification of thymic NENs [8], all tumors except one were ACs in our study. Case #4 had a largely necrotic main tumor corresponding to AC, but in a separate tumor nodule in the close vicinity of the main tumor, mitotic count was 25 per 2 mm^2^, in consistency with LCNEC. Previously, only typical and atypical carcinoids, but no LCNEC or small cell carcinoma, have been reported in the setting of MEN1 [25, 26]. Although this may be partially related to different diagnostic criteria of thymic NENs in earlier literature, the criteria and nomenclature of 2021 WHO classification are the same as in previous 2004 and 2015 editions, and to the best of our knowledge, case #4 is the first MEN1-associated LCNEC reported. It is noteworthy that the two NENs of different grade in this case may represent the ability of MEN1-associated thymic tumors to progress from intermediate- to high-grade, a phenomenon that has been postulated in the lung [27]. However, we cannot exclude the possibility of two synchronous primary tumors.

The Ki-67 index has been used as an ancillary stain, but even in the 2021 WHO classification it is not a formally recognized criterion for grading of either thymic or pulmonary NENs. Furthermore, no reliable cutoff has been established for Ki-67 in the distinction between TC and AC, nor between AC and neuroendocrine carcinomas of either organ. This also applies to the 2018 WHO proposal for a uniform classification of NENs, although reporting the Ki-67 labeling index is advised in the pathology report [24]. In our study, the highest mitotic counts and Ki-67 index were observed in the patient with LCNEC. His serious course of TNET is in accordance with previous data showing that Ki-67 index ≥10 is associated with a poor prognosis [16, 28].

In Finland, TNETs were observed in 3.2% of known MEN1 patients and in only two families in Northern Finland, one of them being a known large pedigree of MEN1 cases. Two types of mutations in the *MEN1* gene (c.1579 C > T and c.1546_1547insC) were observed in our patients. The second one is a known founder mutation in Northern Finland [29, 30]. The alteration of a Cytosine (C) to a Thymine (T) at position 1579 causes a premature stop codon resulting in a strongly truncated protein R527* (–84 amino acids). The insertion of a Cytosine at position 1546 leads to a frameshift which changes an Arginine at codon 516 to a Proline and creates a premature stop codon at position 15 of the new reading frame. This leads to a strongly truncated protein, R516Pfs* (–80 amino acids), which may lead to functional inactivation of menin [31]. In our large pedigree of MEN1, c.1546_1547insC mutations were inherited from both mothers and fathers.

In our series, there were two pairs of TNET-brothers in the large pedigree. The brothers from the most recent generation had earlier penetrance of TNET with a mean difference of 21 years compared to the brothers from the previous sequential generation. Tumor screening and diagnostic methods have evolved between generations in our study leading to possible earlier observations of TNETs in the most recent pair of the brothers. Therefore, the more systematic surveillance with better imaging methods is one explanation in this difference, but it does not seem to be the only explanation of the rather large difference in the observed appearance of TNETs in these pairs of the brothers, especially when considering the poor outcome of these tumors. A genetic anticipation could also been involved. This is a phenomenon where the symptoms of the genetic disorder appear at an earlier age with each generation when this disorder is passed on to the next generation. It has been suggested that the genetic anticipation is a feature explaining the age-related penetrance of some MEN1 linked manifestations [32, 33], such as GEP-NET, PHPT and bpNET [33]. The role and genetic mechanisms involved in possible genetic anticipation of MEN1-TNET in previous studies have been difficult to assess due to its low prevalence and small number of cases. Our results suggest that a genetic anticipation-like earlier disease onset could also be present in some MEN1-TNETs. However, surprisingly, there was not a single case of TNET in our largest Northern Finland MEN1 founder pedigree with tens of family members carrying c.1356_1367del12 (formerly 1466del12) mutations. Therefore, our results should be interpreted with caution.

Somatic *MEN1* mutations have been reported in tumor samples from patients with sporadic TNETs, but have not previously been found in tumor samples of MEN1-associated TNETs [6, 13, 26, 34]. However, a *MEN1* mutation was recently reported in a tumor sample of TNET in a MEN1 family [35] and also in a recent larger study of MEN1-TNETs patients [36]. In two of our patients (#2, #4), we have previously shown that there was no loss of heterozygosity (LOH) in the MEN1 region in 11q13 [6]. Also in the recent NIH study, in two of 12 patients with MEN1-TNETs LOH was not found [36]. Therefore, although the Knudson two-hit hypothesis is involved in the pathogenesis of TNETs in MEN1-patients [36], some other mechanisms are also implicated. Clustering of TNET in some MEN1-families and a strong genetic component in the heritability of MEN1-TNET have previously been shown [37]. Our data confirm propositions that genetic predisposition to TNET in MEN1 is related to the effects of modifier genes and/or some epigenetic factors that modify the expression of *MEN1* gene-related lesions [36–38].

Previous studies have shown that TNET is a serious malignancy [10], and this also applies to MEN1-TNET cases [7, 13, 14]. TNETs, DNETs and PNETs are the main causes of death in MEN1 patients [39, 40]. In our patients, the 5-year survival of the TNET patients was 62.5% and 10-year survival was 31.3%. This is in accordance with previous findings in TNET series [10, 28] and also in TNET series in MEN1 patients [7, 15, 17, 19, 20]. However, in this study, after 5.2 years of follow-up, TNET was the cause of death in only one patient, whereas in 50% of the patients, no metastases were present at the end of the follow-up. The course of the disease and the survival of the patients was better if diagnosis of TNETs was done by CT screening than when apparent clinical symptoms were already present. Large tumor size is an unfavorable risk factor in TNET prognosis [28]. In this study, tumors were smaller when found by screening. This emphasizes the importance of early recognition of new MEN1 cases in MEN1 families and also the value of early identification of small TNETs by their systematic screening in MEN1 patients, especially in MEN1 families with previous TNET cases [31, 37, 41]. Previous studies have shown that aggressive TNET can occur already at the age of 16 years [42]. The earliest age of TNET presentation in the current study was 25 years. Therefore, we consider that it is appropriate to start TNET screening at the age 20 years at the latest [41–43]. CT, MRI and SRS were all valuable imaging tools in this series. In order to minimize the risk of ionizing radiation, chest MRI is probably the most suitable modality for tumor screening of TNETs, at least in the younger group of patients [44].

All our patients were males, which has been a common feature in previous TNET studies in MEN1 [7, 14]. However, TNET in MEN1 can also occur in female patients [7, 15]. Smoking has been a common finding in previous TNET cases in MEN1 [14]. The influence of smoking on TNET pathogenesis is uncertain. Our study with half of the patients being non-smokers shows that appearance of TNET in MEN1 patients is not limited to smokers, as has also been found earlier [15].

Ectopic hormonal syndromes in TNET-associated cases are rare in MEN1 patients [7] compared to sporadic TNET cases [4, 9]. One of our patients (#5) had increased serum IGF-1 levels. Clinical findings did not give rise to a suspicion of active acromegaly. The patient didn’t present any acromegalic features, IGF1 concentration didn’t correlate with tumor size, GH values were suppressed in 3 h oral glucose tolerance test and magnetic resonance imaging revealed only a cystic lesion instead of hypophyseal adenoma or hyperplasia. He also had increased plasma chromogranin A levels without PNET findings. We have previously shown in one PNET-associated acromegalic MEN1-patient with ectopic growth hormone-releasing hormone secretion that SSA treatment can normalize growth hormone levels [45]. Therefore, it is not excluded that SSA treatment in the study patient #5 stabilized the serum IGF-1 levels and prevented the appearance of acromegaly. One case of TNET-associated ectopic growth hormone-releasing hormone secretion has previously been reported in a MEN1 patient [46].

The resectability of TNET is an important factor in prognosis [10]. Radical surgery and a complete resection of TNETs were associated with good survival in this series. Postoperative radiotherapy was given in one patient as a curative-intent treatment after a radical tumor resection, and in two patients with metastases as a palliative-intent treatment. The role of radiotherapy in TNET is controversial [4, 9, 47], and it is uncertain whether this therapy had a significant palliative role in metastasis progression in this study. PRRT was used in one patient as a palliative-intent treatment. After the first four cycles, PRRT did not stabilize the disease progression, but we cannot exclude its positive role in the rather long course of disease in this patient. Previously, heterogeneous responses have been reported with PRRT in TNET patients with MEN1 [48]. SRS was positive in 80% of our patients, as also observed previously [14]. It has been recommended that SSA treatment should be used in SRS positive TNET patients in the initial treatment when there are residual tumor findings post-operatively [9, 49]. In the present study, SSA (octreotide) was used in two patients with metastasis. Minor palliative effects in the course of tumors were possible. Interferon was used in two patients (#1, #4) [50]. Patient #1 used it for renal carcinoma. The role of chemotherapies in TNET is uncertain [9, 16, 51] and in our patients they were ineffective. However, the mTOR inhibitor everolimus seemed to have a significant palliative progression-slowing effect in one patient with extensive metastatic disease. This is in accordance with previously reported findings from the LUNA trial [52].

In conclusion, in our series of MEN1 patients, most TNETs were observed in one large MEN1 founder pedigree, where an anticipation-like earlier disease onset was observed in the most recent generation. Our results confirm previous findings that TNET in MEN1 patients is a serious and aggressive disease. However, the prognosis can be better if TNET is diagnosed in its early course by systematic screening, starting as early as at the age of 20 years. This enables diagnosing smaller tumors that can be easily resected, radical surgery being the main curative therapy in these patients. We have also shown that LCNEC can be associated with TNET in MEN1 patients. The Ki-67 index can be useful prognostic marker for MEN1-associated TNETs. Everolimus seems to have a stabilizing effect in metastatic TNET in MEN1, but patients probably benefit from a tailored approach if the first treatment regimen fails.
